# Asiaticoside improves depressive-like behavior in mice with chronic unpredictable mild stress through modulation of the gut microbiota

**DOI:** 10.3389/fphar.2024.1461873

**Published:** 2024-10-18

**Authors:** Qingyi Ren, Chenxi He, Yuhong Sun, Xiaowei Gao, Yan Zhou, Tao Qin, Zhuo Zhang, Xiaodong Wang, Jun Wang, Siping Wei, Fang Wang

**Affiliations:** ^1^ Pharmaceutical Technology Key Laboratory of Luzhou, Central Nervous System Drug Key Laboratory of Sichuan Province, School of Pharmacy, Southwest Medical University, Luzhou, China; ^2^ Department of Pharmacology, School of Pharmacy, Southwest Medical University, Luzhou, China; ^3^ Department of Hepatobiliary Disease, The Affiliated Traditional Chinese Medicine Hospital, Southwest Medical University, Luzhou, China; ^4^ Key Laboratory for Chemistry and Molecular Engineering of Medicinal Resources (Guangxi Normal University), Guilin, China

**Keywords:** asiaticoside, chronic unpredictable mild stress (CUMS), depression, gut microbiota, microbiota-gut-brain axis, brain-derived neurotrophic factor (BDNF), 5-hydroxytryptamine receptor 1A (5-HT1A)

## Abstract

**Background:**

Asiaticoside, the main active ingredient of Centella asiatica, is a pentacyclic triterpenoid compound. Previous studies have suggested that asiaticoside possesses neuroprotective and anti-depressive properties, however, the mechanism of its anti-depressant action not fully understood. In recent years, a growing body of research on anti-depressants has focused on the microbiota-gut-brain axis, we noted that disruption of the gut microbial community structure and diversity can induce or exacerbate depression, which plays a key role in the regulation of depression.

**Methods:**

Behavioral experiments were conducted to detect depression-like behavior in mice through sucrose preference, forced swimming, and open field tests. Additionally, gut microbial composition and short-chain fatty acid (SCFA) levels in mouse feces were analyzed 16S rRNA sequencing and gas chromatography-mass spectrometry (GC-MS). Hippocampal brain-derived neurotrophic factor (BDNF) and 5-hydroxytryptamine receptor 1A (5-HT1A) expression in mice was assessed by western blotting. Changes in serum levels of inflammatory factors, neurotransmitters, and hormones were measured in mice using ELISA.

**Results:**

This study revealed that oral administration of asiaticoside significantly improved depression-like behavior in chronic unpredictable mild stress (CUMS) mice. It partially restored the gut microbial community structure in CUMS mice, altered SCFA metabolism, regulated the hypothalamic–pituitary–adrenal axis (HPA axis) and inflammatory factor levels, upregulated BDNF and 5-HT1A receptor protein expression, and increased serum serotonin (5-hydroxytryptamine, 5-HT) concentration. These findings reveal that asiaticoside exerts antidepressant effects via the microbiota-gut-brain axis.

**Conclusions:**

These results suggested that asiaticoside exerts antidepressant effects through the microbiota-gut-brain axis in a CUMS mouse model.

## Introduction

Depression is on a significant upward trend, in part due to the growing pressures of rapidly changing socioeconomic pressures, as well as improved diagnosis and reporting, which has accelerated after the 2019 COVID global outbreak. The rates of depression are estimated to affect up to 10% of the global population (https://www.who.int/news-room/fact-sheets/detail/depression), constituting a serious social and medical challenge (Huang et al., 2019; Smith and Mazure, 2021; Goodwin et al., 2022; COVID-19 Mental Disorders Collaborators, 2021; Ettman et al., 2022). Pharmacological therapy, which consists of medications such as selective serotonin reuptake inhibitors (SSRIs), and serotonin-norepinephrine reuptake inhibitors (SNRIs), is commonly used to treat depression. However, due to limited effectiveness and increasing resistance of these antidepressants, they are often inadequate to meet the needs of those suffering from depression. The pathophysiology of depression is extremely complex, and currently recognized hypotheses include monoamine (Hamon and Blier, 2013), neurotrophic factor (Kishi et al., 2017), hypothalamic–pituitary–adrenal (HPA) axis (Keller et al., 2017), and cytokine (Pariante, 2017) hypotheses. While research continues to reveal the underlying mechanisms of depression, the specific pathophysiology remains unknown.

Gut microbiota, which has been extensively researched in recent years, has been established to significantly impact human physiological and pathological behaviors. Dysbiosis of the gut microbiome can cause serious metabolic disorders and diseases (Martin et al., 2018; Pires et al., 2024). Clinical studies and animal model testing have demonstrated that disruption of the gut microbial community structure and diversity can induce or exacerbate depression (Jiang et al., 2015; Naseribafrouei et al., 2014). Simultaneously, improving the state of the gut microbiota can prevent and treat depression (Tillmann et al., 2019; Vuong et al., 2017).

Alterations in the composition of the gut microbiota also change the permeability of the gut barrier (Martel et al., 2022), in which bacteria and bacterial products can be transferred from the gut to the bloodstream, lymph nodes and other organs, to activate a systemic inflammatory response (Madison and Bailey, 2024; Caso et al., 2021; Miller and Raison, 2016), and hypothalamo-pituitary-adrenal axis activity (Osadchiy et al., 2019), reduce the levels of brain-derived neurotrophic factor (BDNF) and finally inducing depression (Du et al., 2020). Notably, short-chain fatty acids (SCFAs), decrease in response to imbalances in the microbiota as a metabolite of the gut microbiota (Koh et al., 2016), which may result in an inflammatory response as well as dysregulation of the HPA axis (Liu et al., 2021; van de Wouw et al., 2018). Acetic acid, propionic acid and butyric acid, the important neuromediators of short-chain fatty acids, may directly affect the brain by crossing the blood-brain barrier (BBB), altering BBB integrity, neurotransmission, neurotrophic factors and serotonin synthesis (Braniste et al., 2014; Varela et al., 2015; Barichello et al., 2015; Morais et al., 2021). So gut microbiome can regulate the gut-brain axis through neuroendocrine, neuroimmune and neural pathways by directly or indirectly producing metabolites of short-chain fatty acids (SCFAs) (Asgari et al., 2024). A new perspective suggests that the microbiota-gut-brain axis may hold the key to understanding depression, and could contribute to the development of novel antidepressant drugs (Chang et al., 2022).

Asiaticoside, the main active consistent of *Centella asiatica* (L.) Urb, is a pentacyclic triterpenoid consistent, has various pharmacological effects such as anti-tumor, neuroprotective, wound healing, and anti-depressant effects (He et al., 2023). Asiaticoside can regulate gut microbiota (Fu et al., 2023). Previous studies have shown that asiaticoside exerts its antidepressant effect by activating brain-derived neurotrophic factor (BDNF) signaling in the hippocampus (Luo et al., 2015), although the exact mechanism remains unknown. Therefore, this study aimed to explore whether the antidepressant effects of asiaticoside are mediated by alterations in the gut microbiome of mice that exhibit depression-like behavior.

## Materials and methods

### Animals

Male C57BL/6 mice (five-to 6-weeks old) were purchased from Luzhou Yinhui Biotechnology Co. LTD., PR China. Each of the six mice was placed individually in a cage (320 × 215 × 170 mm) with a standard 12-h light/dark cycle, where the lights were turned on at 09:00 a.m. Before the experiment began, a 1-week period of adaptive feeding was conducted. Environmental temperature and relative humidity were maintained at 23°C–26°C and 55%, respectively. This study was approved by the Ethics Committee for Laboratory Animals of Southwest Medical University and complied with all relevant ethical guidelines for animal research and human participation (No. swmu20230026).

### Chemicals and antibodies

Asiaticoside (HPLC purity 95%) was purchased from Shanghai Xushuo Biotechnology Co., Ltd. (Catalog No. DC21453-5g). Fluoxetine Hydrochloride (HPLC purity 98%) was purchased from Shanghai Macklin Biochemical Technology Co. Ltd. (Catalog No. F844356-100g). BDNF Polyclonal antibody was purchased from Proteintech Group, Inc. (Catalog No. 28205-1-AP), whereas the Anti-5HT1A Receptor antibody was purchased from Abcam (Catalog No. ab85615). The GAPDH antibody was purchased from Affinity Biosciences (Catalog No. AF7021).

### Drug administration

To evaluate the effects of asiaticoside on depressive mice, the mice were divided into six groups: control (CON), chronic unpredictable mild stress (CUMS), asiaticoside low-dose (AS-L, 10 mg/kg), asiaticoside medium-dose (AS-M, 20 mg/kg), asiaticoside high-dose (AS-H, 40 mg/kg), and fluoxetine (FLX, 20 mg/kg). Asiaticoside and fluoxetine were ultrasonically dispersed in saline containing 0.5% Tween-80 and administered by oral gavage, following the methods described in previous reports (Liang et al., 2008; Wang et al., 2020; Yi et al., 2014). The drug treat was administered once daily for the last 4 weeks of the experiment.

### CUMS depression mouse model

Before the initiation of CUMS treatment, the animals were randomly divided into two groups: stressed and control. Control mice were kept in separate rooms, devoid of any other interference, and were not exposed to stressed mice. Stressed mice were subjected to the CUMS protocol as previously described (Li et al., 2013; Ruilian et al., 2021). Briefly, the weekly stress regimen comprised day and night adjustments, fasting, water deprivation, horizontal shaking, restraint, high and low temperature swimming, a light clip of rat tail, wet litter, tilting squirrel cage, noise, and flash stimulation. All of these stressors were individually and consistently administered both during the day and at night. To prevent the mice from becoming habituated and to ensure the unpredictability of the stressors, all stressors were randomly scheduled over a 1-week period and were repeated throughout the 7-week experiment. Subsequently, sucrose preference, open field, and forced swimming tests were conducted to assess the success of simulating depressive behavior in mice.

### Behavioral tests

#### Sucrose preference test (SPT)

The SPT serves as an important indicator for assessing animal pleasure, with a decrease in pleasure being the primary symptom of depression in mice. When mice are in an anxious or depressive state that leads to a loss of pleasure, they tend to avoid consuming sugary water (Markov, 2022). The SPT was conducted in the 6th week following modeling and in the 11th week following drug administration. Before the test, the mice were trained to adapt to the sucrose solution (1%, w/v) by providing them with two bottles of sucrose solution in each cage for 24 h and then replacing one bottle with water for 24 h. After adaptation, mice were deprived of water and food for 24 h. The test was conducted at 9 a.m. The mice were housed in individual cages and had free access to two bottles: one containing sucrose solution and the other containing water. After 6 h, the positions of the bottles were switched, and after another 6 h, the volumes of sucrose solution and water consumed were recorded. Sucrose preference was calculated as follows: [sucrose solution intake/(sucrose solution + water) intake] × 100%.

#### Forced swimming test (FST)

The FST and OFT are behavioral tests widely used to evaluate depression in mice and the effectiveness of different antidepressant drugs (Nestler and Hyman, 2010). The FST was conducted in the 6th week following modeling and in the 11th week following drug administration. The test was conducted using the same protocol as previously described in detail with minor modifications. Briefly, the mice were individually placed in a glass cylinder (30 cm in height and 15 cm in diameter) filled with 15 cm of water at a temperature of 24°C ± 2°C. The mice were immersed in water for 6 min, with the first 2 min for adaptation and the remaining 4 min to record the immobility time. When the mouse floated upright to keep its head above the water and made only minor movements, it was considered to be immobile. Water was replaced after each trial, and a quiet environment was maintained throughout the experiment.

#### Open-field test (OFT)

Open field experiments were conducted in the 6th week following modeling and in the 11th week following drug administration. The open field experiment was performed as previously described with slight modifications (Cruz et al., 2009; Menneson et al., 2019). The experimental apparatus consisted of a 50 × 50 × 40 cm^3^ cardboard box divided into 25 equal squares (10 × 10 cm^2^) at the bottom. At the beginning of the experiment, a single mouse was placed in one corner of the cardboard box, and its immobility time within 5 min was recorded using a camera, followed by cleaning with 75% ethanol after each test. The experiment was conducted in a quiet environment.

#### Western blotting

After the final behavioral test, all mice were decapitated between 12:00 PM and 2:00 PM. Immediately thereafter, the hippocampal region of the brain was excised and stored at −80°C for subsequent protein level analysis. The hippocampal samples were homogenized in lysis buffer and incubated on ice for 30 min. The homogenates were centrifuged at 14,000 rpm for 20 min at 4°C and the supernatants were collected. The protein concentrations of the samples were determined using BCA assay. Proteins were separated by SDS-PAGE, and the resulting proteins were transferred onto a PVDF membrane. The membranes were blocked in blocking buffer at room temperature for 15 min, and incubated with the appropriate primary antibodies overnight at 4°C (anti-BDNF: 1:1,000, anti-5HT1A:1:1,000, anti-GAPDH: 1:1,000). After three washes with TBST, the membranes were incubated with an HRP-labeled secondary antibody (1:3,000). The blots were washed three times with TBST buffer and immunoreactive bands were detected using an enhanced chemiluminescence technique. The results were normalized to the GAPDH protein expression levels in each sample.

#### ELISA

After the final behavioral test, all mice were decapitated between 12:00 PM and 2:00 PM to avoid hormonal fluctuations, and blood samples were immediately collected. Blood samples were collected on ice and centrifuged at 4°C (12,000 rpm for 10 min) to separate serum. The serum was stored at −20°C for subsequent testing. Serum samples were analyzed using Andygene ELISA kits, including the BDNF (Catalog: AD2815Mo-48T), Cortisol (CORT) (Catalog: AD2093Mo-48T), Corticotropin-releasing Hormone (CRH) (Catalog: AD3341Mo-48T), 5-Hydroxytryptamine (5-HT) (Catalog: AD3398Mo-48T), Mouse IL-6 (Catalog: AD3430Mo), Mouse TNF-α (Catalog: AD3051Mo), and Mouse IL-10 (Catalog: AD2776Mo) ELISA Kits.

### Gut microbiome and metabolomic testing

#### 16S rRNA gene sequencing and data processing

At the end of behavioral testing, mouse fecal samples were collected, placed in sterile tubes, and flash-frozen in liquid nitrogen. The fecal samples were stored in a refrigerator at −80°C until further analysis. Total microbial genomic DNA was extracted from mouse fecal samples using the E. Z.N.A. Stool DNA Kit (OMEGA, Bio-Tek, United States) according to manufacturer’s instructions. The quality and concentration of the DNA were determined using 1.0% agarose gel electrophoresis and a NanoDrop^®^ ND-2000 spectrophotometer (Thermo Scientific Inc., United States). The hypervariable region V3-V4 of the bacterial 16S rRNA gene was amplified using the primer pair 338F (5′-ACT​CCT​ACG​GGA​GGC​AGC​AG-3′) and 806R (5′-GGACTACHVGGGTWTCTAAT-3′). After all samples were amplified in triplicate, the PCR product was extracted using 2% agarose gel, purified, and quantified using the Quantus™ Fluorometer (Promega, United States). After quantification, the purified amplicons were combined in equimolar ratios and paired-end sequenced on an Illumina MiSeq platform (Illumina, San Diego, CA, United States) at Majorbio Bio-Pharm Technology Co., Ltd. (Shanghai, China). Bioinformatic analysis of the gut microbiota community was performed using the free online i-Sanger Cloud Platform of Majorbio (www.i-sanger.com).

#### Determination of short-chain fatty acid (SCFA) levels

The obtained mouse fecal samples were accurately weighed (25 mg) and placed into a 2 mL grinding tube, and 500 µL of water containing 0.5% phosphoric acid was added. The samples were frozen and ground at 50 Hz for 3 min, repeated twice, sonicated for 10 min, and centrifuged at 13,000 g for 15 min at 4°C. To extract the supernatant, 200 µL aqueous solution of the supernatant was placed in a 1.5 mL centrifuge tube, followed by 0.2 mL of n-butyl alcohol solvent containing the internal standard 2-ethylbutyric acid (10 μg/mL). Finally, the samples were vortexed for 10 s, sonicated at a low temperature for 10 min, and centrifuged at 13,000 g for 5 min at 4°C. The supernatant was collected and analyzed using an Agilent 8890B gas chromatograph coupled to an Agilent 5977B/7000D mass-selective detector with an inert electron impact (EI) ionization source with an ionization voltage of 70 eV (Agilent, United States of America). Separations were performed using a HP-FFAP (30 m × 0.25 mm × 0.25 µm) capillary column at a constant flow rate (1 mL/min) using 99.999% helium as the carrier gas. The gas column temperature was set at 80°C, then increased to 120°C at a rate of 40°C/min, 200°C at a rate of 5°C/min, and finally 220°C for 3 min. Samples were injected at a volume of 1 µL in splitting mode (10:1) with an inlet temperature of 180°C. The ion source temperature was 230°C and the quadrupole temperature was 150°C.

#### Statistical analyses

Unless otherwise specified, statistical analyses were performed using GraphPad Prism version 8.0. Multiple group comparisons were assessed using one-way analysis of variance (ANOVA) with Dunnett’s test or *post hoc* Tukey-Kramer test, and differences between each pair of groups were determined using two-tailed Student’s t-test or Wilcoxon rank sum test. All data were considered to be statistically significant at *p* < 0.05.

## Results

### Effect of asiaticoside on depression-like behavior in mice

After exposing the mice to chronic unpredictable stress for 6 weeks, the SPT, FST, and OFT were conducted to assess the development of depression-like symptoms (Supplementary Figure S1). After successful modeling, CUMS mice were randomly divided into six groups and administered daily doses of physiological saline (CUMS), or asiaticoside at concentrations of 10 mg/kg (AS-L), 20 mg/kg (AS-M), 40 mg/kg (AS-H), or 20 mg/kg fluoxetine (FLX). Behavioral tests were conducted 5  weeks post-treatment.

**FIGURE 1:**
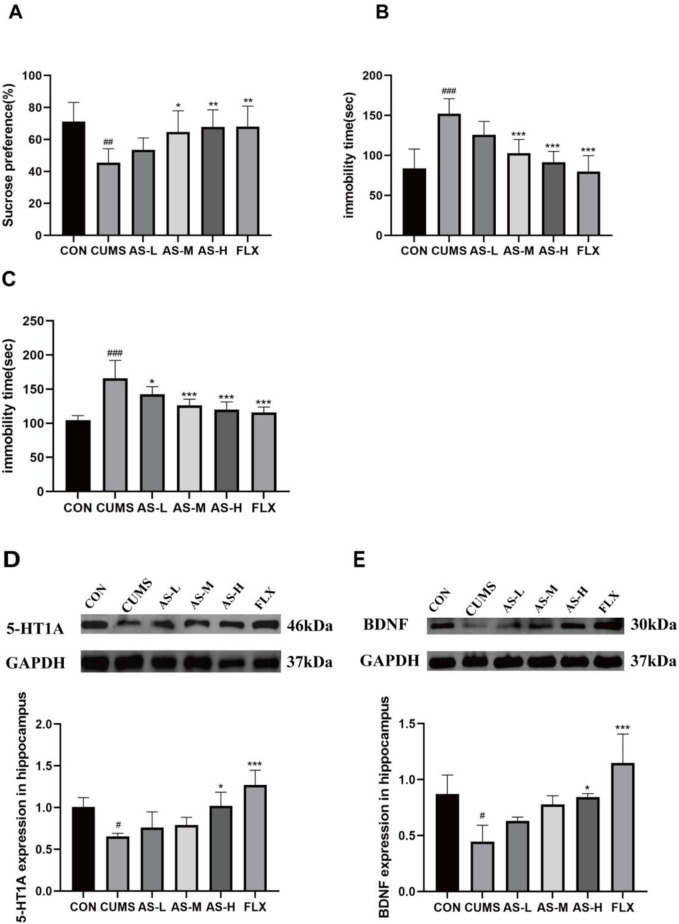
Effects of asiaticoside on sucrose preference **(A)**, FST immobility time **(B)**, and OFT immobility time **(C)** in CUMS mice. Effects of asiaticoside on the expression of 5-HT1A **(D)** and BDNF **(E)** proteins in the hippocampus of CUMS mice (n = 6). Data are expressed as the mean with SD. #*p* < 0.05, ##*p* < 0.01, ### < 0.001 vs. CON group; *p < 0.05, **p < 0.01, ***p < 0.001 vs. CUMS group.

The effect of asiaticoside on sucrose preference in depression-like mice is depicted in Figure 1A. Sucrose preference was significantly lower in the CUMS group than in the CON group. After long-term treatment with asiaticoside and fluoxetine, sucrose preference significantly increased in the AS-M, AS-H, and FLX groups compared to that in the CUMS group. The effect of asiaticoside on FST and OFT immobility time in depressed mice is shown in Figures 1B, C, respectively. Immobility time in the FST and OFT was significantly longer in the CUMS group than in the CON group. After treatment with asiaticoside and fluoxetine, FST immobility time was significantly reduced in the AS-M, AS-H, and FLX groups, whereas OFT immobility time was significantly reduced in the AS-L, AS-M, AS-H, and FLX groups compared to the CUMS group. The SPT, FST, and OFT in the AS-M and AS-H groups showed improvements, especially in AS-H group, which may be attributed to the positive effect of oral asiaticoside on depression-like behavior in mice.

### Effects of asiaticoside on 5-HT1A and BDNF expression in the hippocampus of CUMS mice

The expression levels of 5-HT1A and BDNF in the hippocampus are shown in Figures 1D, E. 5-HT1A and BDNF in the hippocampus were significantly reduced in the CUMS group compared to the CON group. After treatment with asiaticoside and fluoxetine, the AS-H and FLX groups exhibited significantly higher levels of 5-HT1A and BDNF protein expression than the CUMS group. And the expression levels of 5-HT1A and BDNF increased with an increase in the dose of asiaticoside.

### Effects of asiaticoside on gut microbes in mice with depression-like behavior

#### Analysis of intestinal microbial diversity in mice with depression-like behavior

**FIGURE 2:**
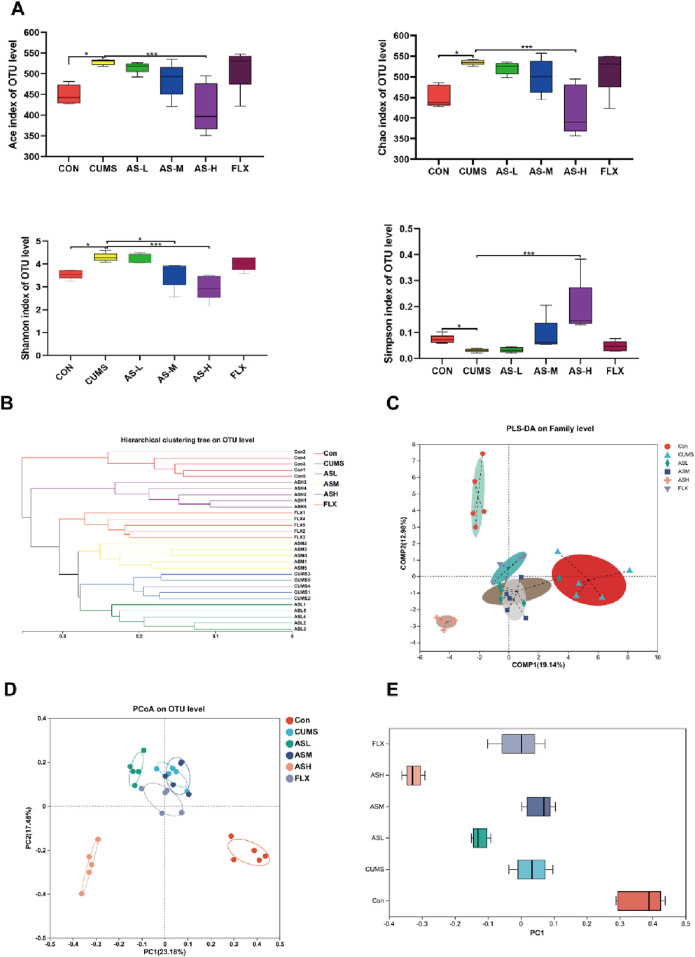
Diversity analysis of the gut microbiota among the six groups. **(A)** α-Diversity indices, that is, Ace and Chao indices reflect microbial community richness, Shannon and Simpson indices reflect microbial community diversity. (*p < 0.05, ***p < 0.001). **(B)** Hierarchical cluster tree of the microbial community in each sample at OTU level. **(C)** PLS-DA of bacterial communities in the six groups at the family level. **(D)** PCoA analysis based on Bray-Curtis distance showing the β-diversity of each group at the OTU level. **(E)** The discrete degree of PC1 is shown in the right pane.

According to the Illumina MiSeq platform (Illumina, San Diego, United States), paired-end sequencing yielded 2,745,161 valid sequences with an average length of 419 nucleotides. Detailed information on the 30 samples is provided in Supplementary Table S1. The rarefaction curve (Supplementary Figure S2) tended to flatten and reach a saturation plateau, indicating that the sequence detection range encompassed most of the microorganisms in the samples. The α-diversity analysis, which was conducted at the operational taxonomic unit (OTU) level using four indices—Ace, Chao, Shannon, and Simpson, revealed the richness and diversity of the gut microbial community in six groups of samples (Figure 2A). The Ace and Chao indices of the CUMS group were significantly higher than those of the CON group, while the Ace and Chao indices of the AS-H group were significantly lower than those of the CUMS group. The Shannon index of the AS-M and AS-L groups was significantly lower than that of the CUMS group, whereas the Simpson index was higher. These findings suggested that the administration of asiaticoside in a dose-dependent manner could alter the richness and diversity of the intestinal flora in mice.

In the β-diversity analysis, the hierarchical clustering tree depicted in Figure 2B demonstrates the relationship between the gut microbiota of the various samples. Each branch of the tree represented the gut microbiota of a sample, and the length of the tree branches represented the distance between samples, with shorter distances indicating higher similarity. The samples of the CUMS group clustered together, far from the CON group, whereas the AS-H group was very close to the CON group, indicating a high similarity between the AS-H and CON groups.

Partial least squares discriminant analysis (PLS-DA) (Figure 2C) indicated that the gut microbial structure of the CUMS group was significantly different from that of the CON group. However, the difference between the AS-M and AS-H groups reduced. The principal coordinates analysis (PCoA) method, which measures β-diversity at the OTU level, effectively differentiated the features of the CON, AS-H, and CUMS groups (Figure 2D), the discrete degree of PC1 is shown in the right panel (Figure 2E), indicating significant structural rearrangement of the gut bacterial community in the CUMS group. In summary, the gut microbial structure of CUMS mice is significantly different from that of normal mice, and when depression-like mice are administered a certain dose of asiaticoside, this difference can be improved, causing the gut microbial community of depression-like mice to resemble that of normal mice.

#### Analysis of gut microbiota composition in different groups

**FIGURE 3:**
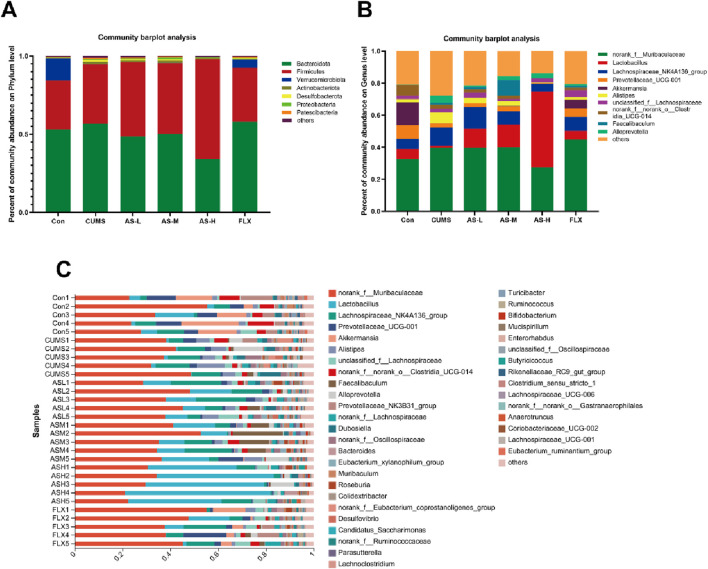
Distribution of bacterial genera and phyla among the six groups. **(A)** Community distribution at the phylum level. **(B, C)** Community distribution at the genus level.

To evaluate the composition of the gut microbiota in each group, we analyzed the abundance of the gut microbial community at the phylum and genus levels. The relative abundance of the gut microbial community at the phylum level (Figure 3A) revealed that Bacteroidetes and Desulfobacterota were more abundant in the CUMS group than in the CON group, although they were less prevalent in the AS-L, AS-M, and AS-H groups. Additionally, the relative abundance of Firmicutes was higher in the AS-H group than that in the CUMS group. Figures 3B, C illustrate the distribution of the gut microbial population at the genus level for each sample, which included 169 bacterial genera. Compared to the CON group, the relative abundance of *Alistipes* and *Alloprevotella* increased in the CUMS group, whereas *Lactobacillus* decreased, and the opposite was observed in the AS group. These findings indicated that depression can alter the gut microbial structure at the phylum and genus levels in mice and that treatment in a dose-dependent manner of asiaticoside could restore the gut microbial structure in depression-like mice.

#### Analysis of differences in gut microbiota in the different groups

**FIGURE 4:**
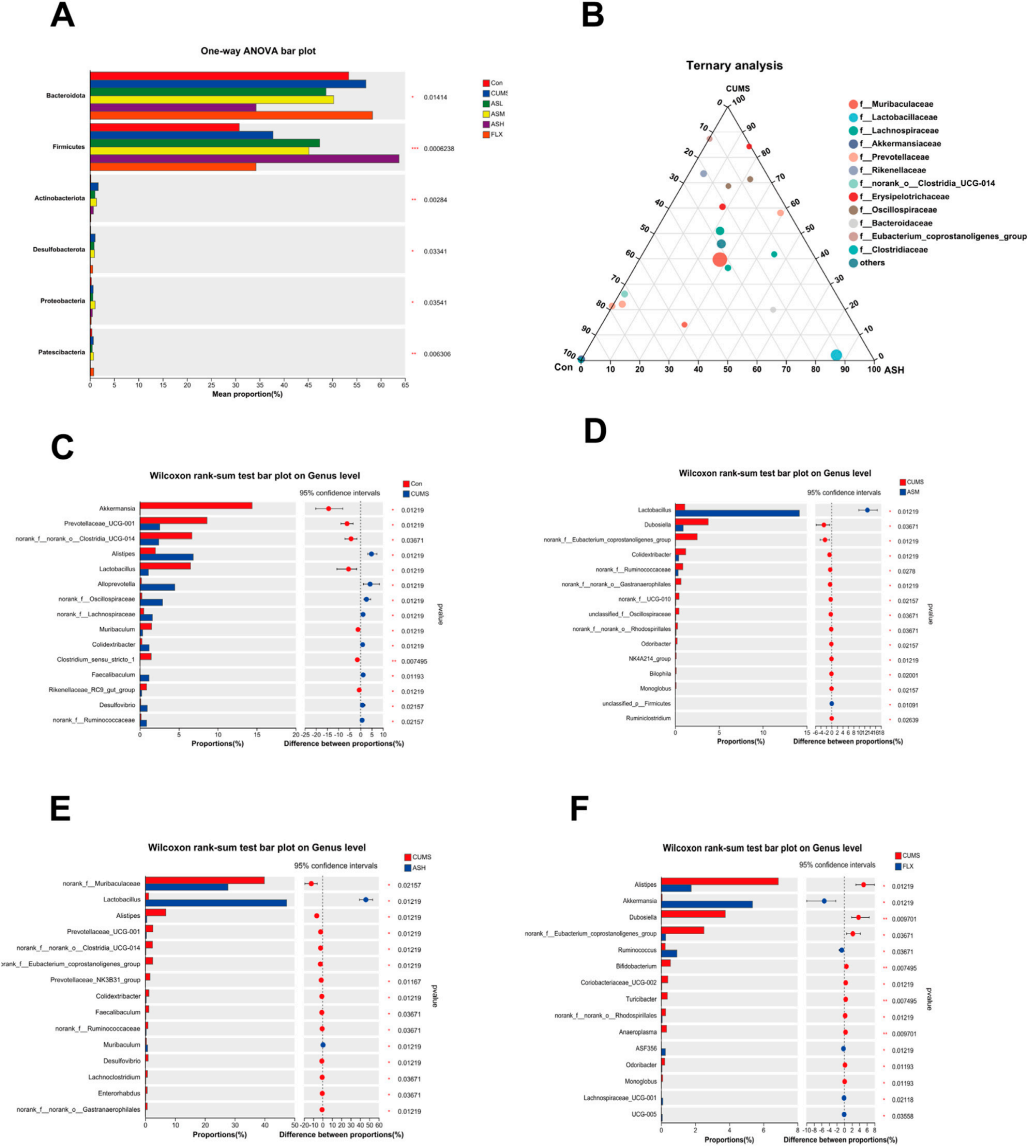
Differences in the relative abundance of specific microbial taxa among the six groups. **(A)** Significant differences in the gut microbiota across the six groups at the phylum level. **(B)** The ternary plot presents the main differences in the gut microbiota in the CON, CUMS, and AS-L groups at the genus level. The dot size indicates the relative abundance of the genera. **(C–F)** Significant differences in gut microbiota between the AS-treated and CUMS groups at the genus level. The Wilcoxon rank-sum test was used for statistical analysis. Data are expressed as mean ± SD. (*p < 0.05, **p < 0.01, and ***p < 0.001).

**FIGURE 5:**
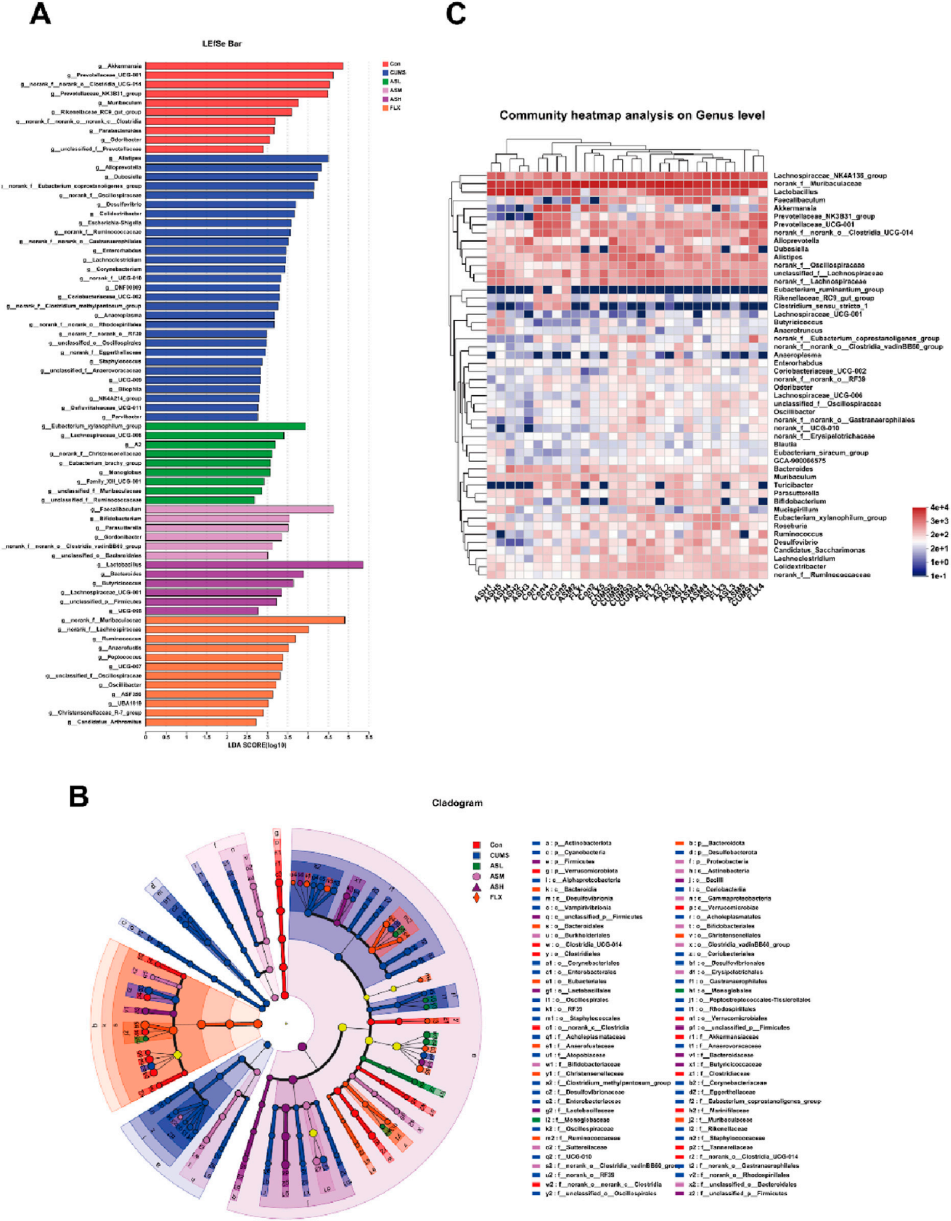
Overall changes in gut bacterial composition among the six groups. **(A)** Linear discriminant analysis (LDA) scores of relative genera in six groups. The length of the column is proportional to the taxa abundance. **(B)** LEfSe analysis of differentially abundant taxa in the six groups. p, phylum; c, class; o, order; f, family; g, genus. **(C)** Heatmap analysis of the bacterial genera in each sample.

As shown in Figure 4A, the relative abundances of *Bacteroidetes, Firmicutes, Actinobacteriota, Desulfobacterota, Proteobacteria*, and *Patescibacteria* demonstrated notable dissimilarity at the phylum level across the six groups. Compared with the CON group, the relative abundances of *Bacteroidetes, Actinobacteriota, Desulfobacterota*, and *Proteobacteria* were significantly higher in the CUMS group and lower in the AS group. Among the groups, the relative abundance of *Firmicutes* was the highest in the AS-L group. The composition and proportional distribution of the dominant genera in the CON, CUMS, and AS-H groups were visually represented in a ternary plot (Figure 4B). Furthermore, the differences in the composition of the gut microbial community between the AS-treated and untreated groups were assessed at the genus level. Compared to the CON group, the abundance of *Akkermansia*, *Prevotellaceae*_*UCG*-*001*, *norank*_*f__norank_o__Clostridia_UCG-014*, *Lactobacillus*, *Muribaculum*, *Clostridium_sensu_stricto_1*, and *Rikenellaceae_RC9*_gut_group were significantly lower in the CUMS group, whereas the abundance of *Alistipes*, *Alloprevotella*, and *Desulfovibrio* was significantly higher (Figure 4C). Compared to the CUMS group, the abundance of *Lactobacillus* was significantly higher in the AS-M group, while the abundance of *Dubosiella*, *Colidextribacter*, *unclassified_f__Oscillospiraceae,* and 13 other genera was significantly lower (Figure 4D). In the comparison between the CUMS and AS-H groups, the abundance of *Lactobacillus* and *Muribaculum* was significantly higher in the CUMS group, whereas the abundance of *norank_f__Muribaculaceae*, *Alistipes*, *Prevotellaceae*_*UCG-001*, *Faecalibaculum*, and *Desulfovibrio*, among the 13 other genera, was significantly lower than that in the CUMS group (Figure 4E). Compared to the CUMS group, the abundance of *Akkermansia*, *Ruminococcus*, and *Lachnospiraceae UCG-001* was higher in the FLX group, while the abundance of *Alistipes*, *Dubosiella*, *norank f Eubacterium*, and *coprostanoligenes_group*, among the 12 other genera, was significantly lower (Figure 4F). To further explore the differences in the gut microbial community among the six groups, LEfSe and hierarchical clustering heatmap (Figures 5A, B) analysis were conducted to reveal specific bacteria and changes in the overall gut microbial community.

#### Effects of asiaticoside on gut metabolite SCFAs in CUMS mice

**FIGURE 6:**
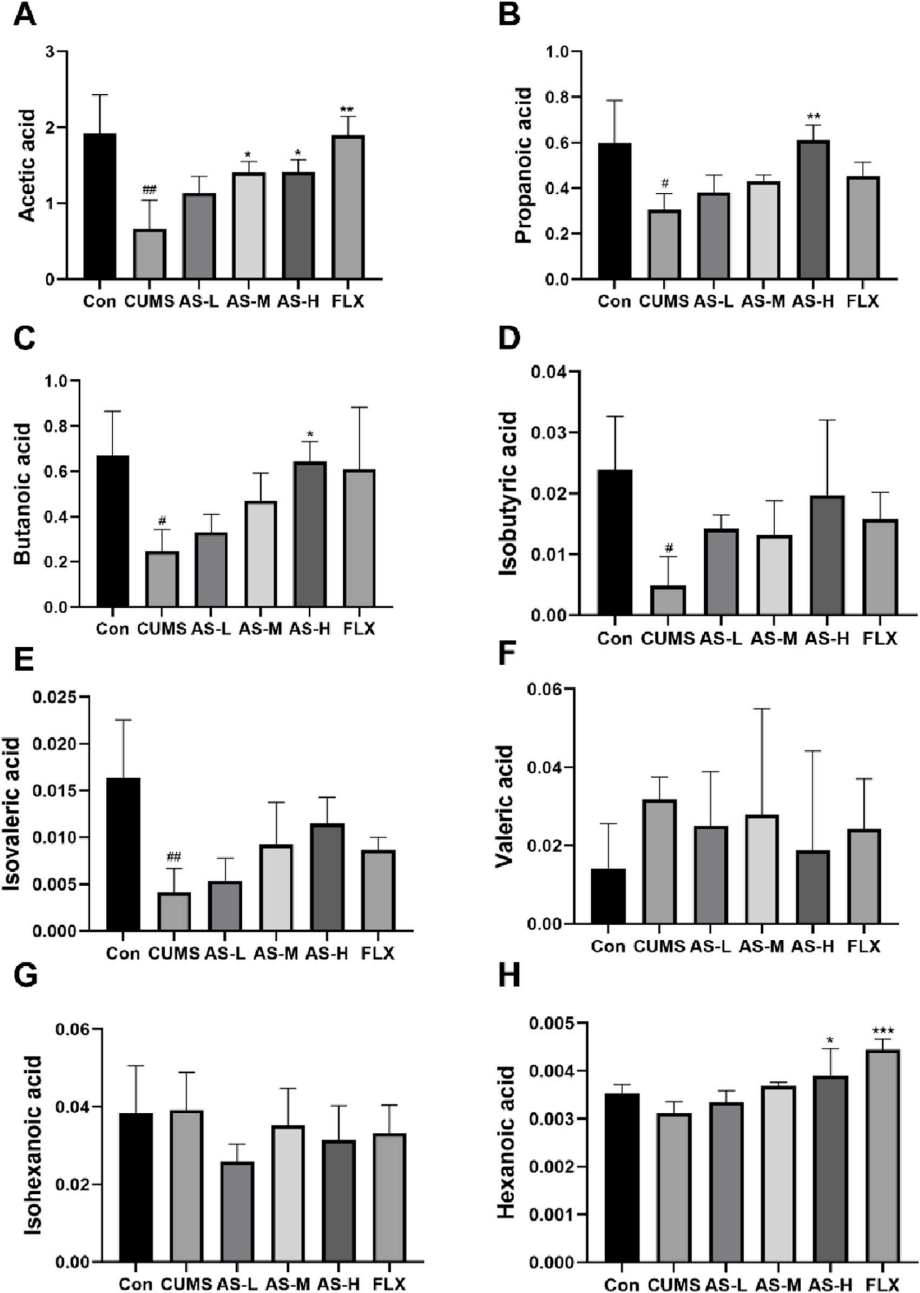
The concentrations (µg/mL) of acetic **(A)**, propanoic **(B)**, butanoic **(C)**, isobutyric **(D)**, isovaleric **(E)**, valeric **(F)**, isohexanoic **(G)**, and hexanoic acid **(H)** in the fecal samples among the six groups were determined using GC-MS. Data are expressed as mean ± SD. #*p* < 0.05, ##*p* < 0.01, ### < 0.001 vs. CON group; *p < 0.05, **p < 0.01, ***p < 0.001 vs. CUMS group.

Gas chromatography-mass spectrometry (GC-MS) was used to evaluate SCFA levels in the feces of mice from six different groups, including acetic acid, propanoic acid, butanoic acid, isobutyric acid, isovaleric acid, valeric acid, isohexanoic acid, and hexanoic acid. Compared to the CON group, the acetic acid content in the CUMS group was significantly reduced, while the AS-M, AS-H, and FLX groups showed a significant dose-dependent increase in acetic acid content compared to the CUMS group (Figure 6A). Additionally, propanoic acid (Figure 6B) and butanoic acid (Figure 6C) contents in the AS-H group were significantly higher than those in the CUMS group. The isobutyric acid (Figure 6D) and isovaleric acid (Figure 6E) contents in the CUMS group were significantly lower than those in the CON group, with the AS group showing an increasing trend compared to the CUMS group. The hexanoic acid content in the AS-H and FLX groups was significantly higher than that in the CUMS group, but there was no significant difference in the hexanoic acid content between the CUMS and CON groups (Figure 6H). There was no significant difference in the contents of valeric acid (Figure 6F) and isohexanoic acid (Figure 6G) among the six groups.

#### Effects of asiaticoside on serum neurotransmitters and inflammatory factors in CUMS mice

**FIGURE 7:**
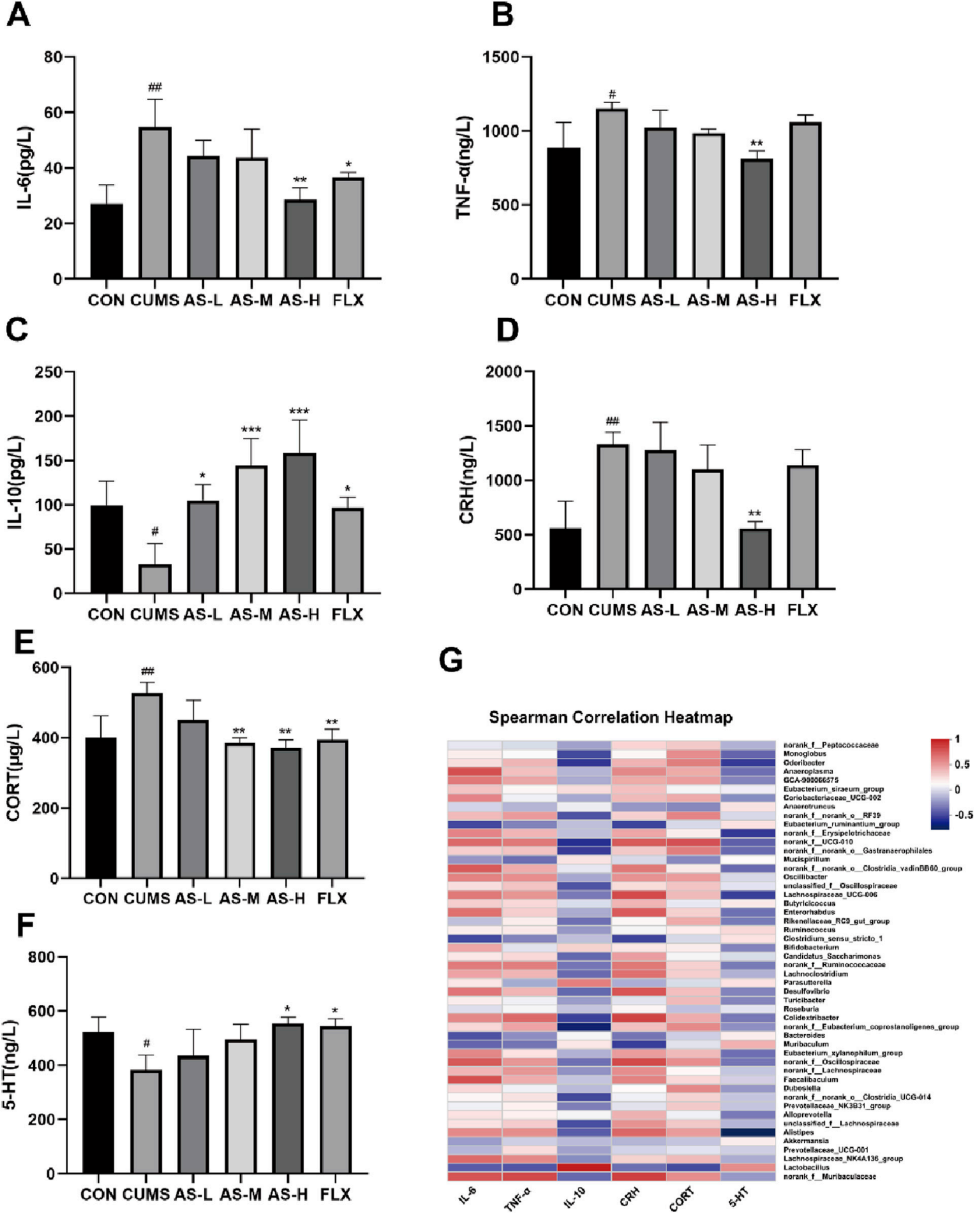
The concentrations of IL-6 **(A)**, TNF-α **(B)**, IL-10 **(C)**, CRH **(D)**, CORT **(E)**, and 5-HT **(F)** in the serum samples were determined using ELISA. Data are expressed as the mean ± SD. *p* < 0.05 was considered statistically significant. #*p* < 0.05, ##*p* < 0.01 vs. CON group; *p < 0.05, **p < 0.01, ***p < 0.001 vs. CUMS group. **(G)** Spearman Correlation H.eatmap.

The correlation between depression and neurotransmitters as well as inflammatory factors is well established. Therefore, in this study, ELISA was used to measure the levels of serum inflammatory factors and neurotransmitters. The levels of IL-6 (Figure 7A) and TNF-α (Figure 7B) were significantly higher in the CUMS group than in the CON group, whereas the levels of IL-10 (Figure 7C) were significantly lower. The levels of IL-6 and TNF-α were significantly lower in the AS group than in the CUMS group, whereas the levels of IL-10 were significantly higher. The effects of asiaticoside on serum CRH and CORT levels are shown in Figures 7D, E. CRH and CORT levels in the CUMS group were significantly higher than those in the CON group, but treatment with asiaticoside or fluoxetine resulted in a significant reduction in these levels. Serotonin (5-HT) plays a key role in the onset and treatment of depression. Our results (Figure 7F) showed that the serum 5-HT levels in the CUMS group were significantly lower than those in the CON group, whereas the 5-HT levels in the AS-H and FLX groups were significantly higher than those in the CUMS group.

#### Sequencing results correlated with changes in neurotransmitter and inflammatory factor levels

The Spearman correlation heatmap (Figure 7G) indicated that higher levels of inflammation (TNF-α and IL-6) and hormone levels (CRH and CORT) were positively correlated with *norank_f__Muribaculaceae*, *Alistipes*, and *Desulfovibrio*, whereas they were negatively correlated with *Lactobacillus*. Additionally, the increases in IL-10 and 5-HT were positively correlated with *Lactobacillus* and negatively correlated with *norank_f__Muribaculaceae*, *Alistipes*, and *Desulfovibrio*.

## Discussion

The findings of this study suggest that asiaticoside can reverse depression-like behavior in CUMS mice by improving gut microbiota, SCFA levels, HPA axis hormone levels, serum inflammatory factors, and protein expression of hippocampal 5-HT1A and BDNF. These results imply that asiaticoside exerts antidepressant effects through restoration of depressive-like behaviors in mice with dysregulated gut microbiota, and dysfunction of many biochemical factors that are critical for the development of depression.

Asiaticoside is a triterpenoid consistent extracted from *Centella asiatica* that exhibits antitumor, anti-inflammatory, and neuroprotective effects (Sun et al., 2020). This study primarily focused on its antidepressant effects. Previous studies have demonstrated that asiaticoside improves depression-like behavior in mice by upregulating 5-HT and BDNF levels in the hippocampus (Wang et al., 2020). Moreover, asiaticoside can upregulate the expression of BDNF, PSD-95, and synapsin I in the hippocampus of CUMS mice. BDNF can potentiate excitatory synaptic transmission and proteins in neurons and the brain, and the expression of PSD95 and synapsin I, as downstream proteins of BDNF, were regulated by BDNF (Luo et al., 2015). Thus, asiaticoside may exert its antidepressant effects by activating BDNF signaling in the hippocampus as well as upregulating 5-HT expression. However, asiaticoside, a triterpenoid compound with a high molecular weight, is not easily absorbed through biological membranes. Consequently, further research is necessary to understand the mechanisms underlying the antidepressant effects of oral asiaticoside administration.

The CUMS mouse model is a commonly used depression model that induces depressive like behaviour through long-term unpredictable physical stress, effectively mimicking various psychological stressors encountered in daily human life (Qiao et al., 2016; Willner, 1997). In this study, administration of asiaticoside and fluoxetine to CUMS mice resulted in an increased sugar water preference rate and a decrease in immobility time during the FST and OFT, suggesting behavioral normalization in the mice. These findings imply that asiaticoside possesses properties and can improve the depressive state in mice.

Furthermore, reduced expression of BDNF in the hippocampus is considered a significant mechanism for the onset of depression (Adachi et al., 2017; Arumugam et al., 2017; Castrén and Monteggia, 2021). This is because CUMS can significantly increase hippocampal autophagy, reduce hippocampal BDNF expression, and induce depressive behavior in experimental animals (Rana et al., 2021). BDNF belongs to the neurotrophic factor family and is distributed in both the brain and periphery. Moreover, it is primarily expressed in the hippocampus and cortex, where it is produced by neurons and astrocytes (Kang et al., 2016). In this study, asiaticoside and fluoxetine reversed the decrease in hippocampal BDNF levels in CUMS mice and improved depressive symptoms.

The monoamine neurotransmitter hypothesis, which posits that alterations in the levels of monoamine neurotransmitters such as 5-HT can contribute to depression, is widely recognized as a key factor in the pathogenesis of depression. 5-HT, a monoamine neurotransmitter, plays a crucial role in regulating behavior, emotions, and memory in the human body and is closely associated with nervous system disorders (Ma et al., 2023). SSRIs, such as fluoxetine, are currently widely used as antidepressant drugs in clinical practice. Studies have shown (Chen et al., 2023) that following the use of fluoxetine, serum 5-HT levels in depressed mice significantly increased, and depressive symptoms improved significantly. In this study, we found that CUMS mice treated with asiaticoside or fluoxetine exhibited an increase in serum 5-HT levels and an improvement in depression, further confirming that asiaticoside is effective in ameliorating the depressive state. Furthermore, the expression of 5-HT1A in the hippocampus of mice was detected. The results revealed that after a period of CUMS, the expression of 5-HT1A in the mouse hippocampus significantly decreased, which is consistent with previous findings (Drevets et al., 2007). Interestingly, after oral administration of asiaticoside or fluoxetine to CUMS mice for a certain period, the expression of 5-HT1A in the hippocampus significantly increased. The 5-HT1A receptor is the most widely distributed subtype of 5-HT receptor in the brain, especially in the hippocampus and prefrontal cortex, and is an important target for the clinical treatment of depression and anxiety disorders (Yohn et al., 2017). Most studies have shown that chronic stress can cause a decrease in hippocampal 5-HT levels, downregulation of 5-HT1A receptors, and a decrease in the function of the 5-HT system (Drevets et al., 2007).

These findings support the antidepressant effect of asiaticoside. However, owing to its poor absorption in the intestinal tract, exerting its antidepressant effects through direct absorption is challenging. In recent years, the development and promotion of high-throughput sequencing have provided a better understanding of the relationship between neurological diseases and microorganisms (Gilbert et al., 2018). The “microbiota-gut-brain axis” has received increasing attention, and there is increasing evidence that the gut microbiota of depressed patients is dysregulated (Chang et al., 2022). Currently, the brain-gut-microbiota axis is not merely a concept, but also represents the interactions between the host’s gut-brain-microbiota system, central nervous system (CNS), endocrine chemical signaling system, and brain and gut barrier function (Dinan and Cryan, 2013).

Therefore, we focused our attention on the gut microbiota. Previous literature suggests that certain traditional Chinese medicine consistents, such as ginsenoside R1, resveratrol, and albiflorin, possess antidepressant properties (Jiang et al., 2020; Zhao et al., 2018; Nabavi et al., 2017). Among these, albiflorin can alleviate depression by regulating the composition of the gut microbiota. However, research on the influence of asiaticoside on the gut microbiota is limited. In this study, we compared the gut microbial community in healthy mice and depression-like mice using the alpha diversity index, PCoA, and hierarchical clustering tree methods. Our findings indicated that the species richness and diversity of the gut microbial community in CUMS mice were higher than those in healthy mice, which contradicts the results of McGaughey and Li (McGaughey et al., 2019; Li et al., 2018). Some studies have also shown that there is no apparent correlation between gut microbial community diversity and depression (Rincel et al., 2019; Zhu et al., 2019), further research is necessary to elucidate the specific role of gut microbial diversity in depression. In this study, oral administration of asiaticoside with increased dose partially restored gut microbial community diversity and species differences, upregulated hippocampal 5-HT1A and BDNF expression, and improved depressive behaviors in mice with depressive-like behaviors.

In the present study, the relative abundances of *Bacteroidetes, Actinobacteriota, Desulfobacterota,* and *Proteobacteria* at the phylum level in CUMS mice were higher than those in normal mice, whereas their abundance was significantly reduced after oral administration of asiaticoside or fluoxetine. The relative abundance of *Firmicutes* in CUMS mice that received a high dose of asiaticoside was significantly increased. In other reports (Li et al., 2018; Yang et al., 2020), the relative abundance of *Bacteroidetes, Actinobacteriota* and *Desulfobacterota* was significantly increased in the gut of patients or mice with depression, further demonstrating that the gut microbial composition of mice exhibiting depression-like behavior was altered. This alteration leads to an increase in intestinal permeability on the one hand and affects the integrity of the blood-brain barrier (BBB) on the other hand (Martel et al., 2022; Braniste et al., 2014; Conn et al., 2024), such that some inflammatory factors can be transferred from the intestinal to the bloodstream and then across the BBB, thereby activated the systemic inflammatory response (Madison and Bailey, 2024). These cytokines, including IL-6 and TNF-α, are also effective activators of the HPA axis (Bodemeier Loayza Careaga and Wu, 2024). Furthermore, gut microbiota -mediated inflammation is strongly associated with the development of depression through the downregulation of BDNF expression involved in NF-κB activation (Han et al., 2021). This is confirmed by our detection of increased serum IL-6, TNF-α, CRH, and CORT levels as well as decreased hippocampal BDNF expression in depression-like mice, these results are both caused by depression and in turn can exacerbate depression. So the administration of asiaticoside to depression-like mice restored the composition of the gut microbiota, could improve intestinal permeability and blood-brain barrier integrity, then could decrease the levels of IL-6, TNF-α, CRH, and CORT and increase hippocampal BDNF levels.

Analysis of the gut microbiota at the genus level revealed that the relative abundance of *Alistipes* and *Desulfovbrio* in CUMS mice was significantly higher than that in healthy mice. However, this condition improved after treatment with high-dose asiaticoside or fluoxetine. *Alistipes,* which belongs to the *Bacteroidetes* family, may be associated with inflammatio (Saulnier et al., 2011; Frémont et al., 2013; Verstreken et al., 2012; Rowan et al., 2009; Dostal Webster et al., 2019). In addition, Jiang et al. found that the relative abundance of *Alistipes* in the feces of patients with depression was also significantly increased (Vuong et al., 2017). *Alistipes* contains tryptophanase, which metabolizes tryptophan to indole. This process affects tryptophan metabolism, which in turn affects 5-HT levels. Inflammation is an essential pathogenic mechanism of depression (Guo et al., 2018). This also explains the increase in 5-HT content, decrease in pro-inflammatory factors TNF-α and IL-6 content, and increase in anti-inflammatory factor IL-10 content in the serum of mice with depression-like behavior after oral administration of asiaticoside. In addition to the increased relative abundance of harmful bacteria in mice with depression-like behavior, the relative abundance of beneficial bacteria was decreased in these mice. Lachnospiraceae_NK4A136_group as a bacterium negatively correlated with intestinal permeability (Yang et al., 2024), its abundance is reduced in depression-like mice, may result in the progression of depression through systemic inflammation caused by increased intestinal permeability. *Lactobacillus* is a bacterial species that belongs to the phylum *Firmicutes*. Studies have shown that oral administration of *Lactobacillus* can improve the depressive state in mice and has an antidepressant effect (Bravo et al., 2011). Specifically, *Lactobacillus* supplementation increased hippocampal BDNF levels (Yun et al., 2023), *Lactobacillus* has been shown to stimulate anti-inflammatory cytokines, including IL-10, and downregulate pro-inflammatory cytokines (Sokol et al., 2008; Llopis et al., 2009). According to our results, the relative abundance of *Lactobacillus* in CUMS mice was significantly lower than that in normal mice, and the relative abundance of *Lactobacillus* in CUMS mice was significantly increased after asiaticoside administration. Therefore, at the genus level, asiaticoside also exhibits a strong ability to restore gut microbial composition in depressed mice.

The primary metabolites of the gut microbiota are SCFAs that can directly affect the brain by crossing the blood-brain barrier (BBB). They can also influence the integrity of the BBB, neurotransmission, biosynthesis of BDNF, and serotonin levels, which are of great significance in regulating depression (Agirman and Hsiao, 2021; Xiao et al., 2020). Our experimental results indicated that SCFA levels in CUMS mice differed significantly from those in normal mice, with a notable decrease in the concentrations of acetic acid, butyric acid, and propionic acid, which presumably caused by disordered gut microbiota in CUMS mice, as *Firmicutes* bacteria can produce short-chain fatty acids (Tian et al., 2022; Samuel et al., 2008). And the relative abundance of *Firmicutes* were decreased in CUMS mice, leading to the speculation that the relative abundance of Firmicutes is positively correlated with the production of acetic, butyric, and propionic acids. In addition, Research has indicated that SCFAs are involved in digestion, immune function, and CNS function. The symptoms of depression in mice can be alleviated by controlling the three most abundant SCFAs: propionic acid, acetic acid, and butyric acid (van de Wouw et al., 2018). Butyric acid can increase the concentration of the central neurotransmitter 5-HT and promote BDNF expression in the hippocampus (Sun et al., 2016). Propionic acid also affects neurotransmission in the brain by interfering with synaptic neurotransmitter release (Jesulola et al., 2018). This suggests that asiaticoside can regulate the gut microbiota of mice with depression-like behavior, thereby influencing the synthesis and metabolism of SCFAs and thus exerting an antidepressant effect by up-regulating the expression of 5-HT and BDNF.

Hypothalamic-pituitary-adrenal (HPA) axis dysregulation is one of the most consistent pathophysiologic manifestations in depression (Pereira et al., 2024). The HPA axis, as a crucial component of the gut-brain axis, may affect the structure of the gut microbiota in animal models in response to various stressors (Bailey and Coe, 1999; Babaei et al., 2022). However, the relationship between gut microbiota and the HPA axis is bidirectional. Studies have shown that probiotics based on *Lactobacillus* and *Bifidobacterium* can restore stress-induced HPA axis dysfunction and improve learning, memory, depression, and anxiety-like symptoms (Desbonnet et al., 2010; Eutamene et al., 2007). Prolonged exposure to unpredictable physical stress in mice can lead to dysregulation of the endocrine system, such as hyperactivation of the HPA axis, which increases the secretion of cortisol (CORT), adrenocorticotropic hormone (ACTH), and corticotropin-releasing hormone (CRH) and which can trigger harmful systemic biological responses and depression (Aas et al., 2019). Studies have shown that chronic CORT can induce depression-like behavior in mice and reduce the expression of BDNF in dentate gyrus (DG) neurons of the hippocampus (Zhang et al., 2023). After treatment with asiaticoside, CUMS mice showed improved depression-like behaviors due to remodeled gut microbiota structure, significant increase in relative abundance of Lactobcillus, suppression of HPA axis hyperactivity, upregulation of hippocampal BDNF expression, and decrease in serum inflammatory factor levels. In addition to the gut microbiota, SCFAs can also regulate the HPA axis. Studies have found that acetic acid directly interacts with the HPA axis in the brain, thereby improving overactivation of the HPA axis (Frost et al., 2014). This suggests that asiaticoside exerts its antidepressant effects by regulating dysregulated acetic acid production and the HPA axis. This bidirectional regulation is also reflected by the HPA axis and inflammatory factors. Alterations in the gut microbiota and overactivation of the HPA axis may contribute to the enhanced release of cytokines and the synthesis of bioactive molecules. Conversely, certain cytokines such as IL-6 and TNF-α may pass through the BBB and activate the HPA axis (Banks, 2005; Hassamal, 2023).

The results of this study demonstrate that asiaticoside can improve the gut microbial structure in CUMS mice, alter SCFA metabolism, regulate the HPA axis and inflammatory factor levels, upregulate hippocampal BDNF and 5-HT1A protein expression, and increase serum 5-HT concentration, thereby improving depression-like behavior in CUMS mice. However, no dose-response trend was observed for asiaticoside at the three doses in Western blot, ELISA, SCFAs, and intestinal microbial analysis. There is currently no clear explanation for this observation. Based on the results of this study, we hypothesize that the optimal dosage of asiaticoside on depression-like mice is 20–40 mg/kg.

## Conclusion

The purpose of this study was to explore whether asiaticoside could ameliorate CUMS-induced depression-like symptoms and its possible mechanism through the microbe-gut-brain axis. Male mice were subjected to CUMS and treated with asiaticoside. Depression-like behavior was measured by a series of behavioral tests. Gut microbiota, short-chain fatty acids, 5-HT1A, BDNF, serum inflammatory factors, and HPA axis-secreted hormones were evaluated. Asiaticoside intervention significantly attenuated CUMS-induced depressive-like behavior in mice due to upregulation of BDNF and 5-HT1A expression in the hippocampus and 5-HT in serum, and downregulation of IL-6, TNF-α, CRH, and CORT. Changes in these metrics were associated with short-chain fatty acids, *Alistipes*, *Desulfovbrio*, *Lachnospiraceae_NK4A136_group*, *Lactobcillus*, and *Firmicute*. Taken together, our results suggest that asiaticoside has a positive ameliorative effect on CUMS mice, which we hypothesized may be due to the restoration of gut microbiota allowing its metabolic pathways to increase the content of short-chain fatty acids, reduce inflammatory responses, inhibit HPA axis hyperactivity, and subsequently stimulate the expression of cerebral BDNF and 5-HT1A.

## Data Availability

The datasets presented in this study can be found in online repositories. The names of the repository/repositories and accession number(s) can be found in the article/Supplementary Material.

## Abbreviations

AS-L, asiaticoside low-dose; AS-M, asiaticoside medium-dose; AS-H, asiaticoside high-dose; BDNF, brain-derived neurotrophic factor; CNS, central nervous system; CUMS, chronic unpredictable mild stress; CORT, Cortisol; CRH, Corticotropin-releasing Hormone; FLX, fluoxetine; HPA, hypothalamic–pituitary–adrenal; SCFA, short-chain fatty acid.

## References

[B1] Aas M. Pizzagalli D. A. Laskemoen J. F. Reponen E. J. Ueland T. Melle I. (2019). Elevated hair cortisol is associated with childhood maltreatment and cognitive impairment in schizophrenia and in bipolar disorders. Schizophrenia Res. 213, 65–71. 10.1016/j.schres.2019.01.011 30660575

[B2] Adachi M. Autry A. E. Mahgoub M. Suzuki K. Monteggia L. M. (2017). TrkB signaling in dorsal raphe nucleus is essential for antidepressant efficacy and normal aggression behavior. Neuropsychopharmacology 42 (4), 886–894. 10.1038/npp.2016.201 27634357 PMC5312065

[B3] Agirman G. Hsiao E. Y. (2021). SnapShot: the microbiota-gut-brain axis. Cell 184 (9), 2524–2524.e1. 10.1016/j.cell.2021.03.022 33930299

[B4] Arumugam V. John V. S. Augustine N. Jacob T. Joy S. M. Sen S. (2017). The impact of antidepressant treatment on brain-derived neurotrophic factor level: an evidence-based approach through systematic review and meta-analysis. Indian J. Pharmacol. 49 (3), 236–242. 10.4103/ijp.IJP_700_16 29033483 PMC5637134

[B5] Asgari R. Bazzazan M. A. Karimi Jirandehi A. Yousefzadeh S. Alaei M. Keshavarz Shahbaz S. (2024). Peyer's Patch: possible target for modulating the Gut-Brain-Axis through microbiota. Cell. Immunol. 401-402, 104844. 10.1016/j.cellimm.2024.104844 38901288

[B6] Babaei F. Mirzababaei M. Mohammadi G. Dargahi L. Nassiri-Asl M. (2022). Saccharomyces boulardii attenuates lipopolysaccharide-induced anxiety-like behaviors in rats. Neurosci. Lett. 778, 136600. 10.1016/j.neulet.2022.136600 35358641

[B7] Bailey M. T. Coe C. L. (1999). Maternal separation disrupts the integrity of the intestinal microflora in infant rhesus monkeys. Dev. Psychobiol. 35 (2), 146–155. 10.1002/(sici)1098-2302(199909)35:2<146::aid-dev7>3.0.co;2-g 10461128

[B8] Banks W. A. (2005). Blood-brain barrier transport of cytokines: a mechanism for neuropathology. Curr. Pharm. Des. 11 (8), 973–984. 10.2174/1381612053381684 15777248

[B9] Barichello T. Generoso J. S. Simões L. R. Faller C. J. Ceretta R. A. Petronilho F. (2015). Sodium butyrate prevents memory impairment by Re-establishing BDNF and GDNF expression in experimental pneumococcal meningitis. Mol. Neurobiol. 52 (1), 734–740. 10.1007/s12035-014-8914-3 25284351

[B10] Bodemeier Loayza Careaga M. Wu T. J. (2024). Chronically stressed male and female mice show a similar peripheral and central pro-inflammatory profile after an immune challenge. PloS one 19 (2), e0297776. 10.1371/journal.pone.0297776 38381770 PMC10880960

[B11] Braniste V. Al-Asmakh M. Kowal C. Anuar F. Abbaspour A. Tóth M. (2014). The gut microbiota influences blood-brain barrier permeability in mice. Sci. Transl. Med. 6 (263), 263ra158. 10.1126/scitranslmed.3009759 PMC4396848 25411471

[B12] Bravo J. A. Forsythe P. Chew M. V. Escaravage E. Savignac H. M. Dinan T. G. (2011). Ingestion of Lactobacillus strain regulates emotional behavior and central GABA receptor expression in a mouse via the vagus nerve. Proc. Natl. Acad. Sci. U. S. A. 108 (38), 16050–16055. 10.1073/pnas.1102999108 21876150 PMC3179073

[B13] Caso J. R. MacDowell K. S. González-Pinto A. García S. de Diego-Adeliño J. Carceller-Sindreu M. (2021). Gut microbiota, innate immune pathways, and inflammatory control mechanisms in patients with major depressive disorder. Transl. psychiatry 11 (1), 645. 10.1038/s41398-021-01755-3 34934041 PMC8692500

[B14] Castrén E. Monteggia L. M. (2021). Brain-derived neurotrophic factor signaling in depression and antidepressant action. Biol. psychiatry 90 (2), 128–136. 10.1016/j.biopsych.2021.05.008 34053675

[B15] Chang L. Wei Y. Hashimoto K. (2022). Brain-gut-microbiota axis in depression: a historical overview and future directions. Brain Res. Bull. 182, 44–56. 10.1016/j.brainresbull.2022.02.004 35151796

[B16] Chen Z. Gu J. Lin S. Xu Z. Xu H. Zhao J. (2023). Saffron essential oil ameliorates CUMS-induced depression-like behavior in mice via the MAPK-CREB1-BDNF signaling pathway. J. Ethnopharmacol. 300, 115719. 10.1016/j.jep.2022.115719 36126781

[B17] Conn K. A. Borsom E. M. Cope E. K. (2024). Implications of microbe-derived ɣ-aminobutyric acid (GABA) in gut and brain barrier integrity and GABAergic signaling in Alzheimer's disease. Gut microbes 16 (1), 2371950. 10.1080/19490976.2024.2371950 39008552 PMC11253888

[B18] COVID-19 Mental Disorders Collaborators (2021). Global prevalence and burden of depressive and anxiety disorders in 204 countries and territories in 2020 due to the COVID-19 pandemic. Lancet London, Engl. 398 (10312), 1700–1712. 10.1016/S0140-6736(21)02143-7 PMC8500697 34634250

[B19] Cruz S. L. Soberanes-Chávez P. Páez-Martinez N. López-Rubalcava C. (2009). Toluene has antidepressant-like actions in two animal models used for the screening of antidepressant drugs. Psychopharmacology 204 (2), 279–286. 10.1007/s00213-009-1462-2 19151967

[B20] Desbonnet L. Garrett L. Clarke G. Kiely B. Cryan J. F. Dinan T. G. (2010). Effects of the probiotic Bifidobacterium infantis in the maternal separation model of depression. Neuroscience 170 (4), 1179–1188. 10.1016/j.neuroscience.2010.08.005 20696216

[B21] Dinan T. G. Cryan J. F. (2013). Melancholic microbes: a link between gut microbiota and depression? Neurogastroenterol. Motil. 25 (9), 713–719. 10.1111/nmo.12198 23910373

[B22] Dostal Webster A. Staley C. Hamilton M. J. Huang M. Fryxell K. Erickson R. (2019). Influence of short-term changes in dietary sulfur on the relative abundances of intestinal sulfate-reducing bacteria. Gut microbes 10 (4), 447–457. 10.1080/19490976.2018.1559682 30810441 PMC6748593

[B23] Drevets W. C. Thase M. E. Moses-Kolko E. L. Price J. Frank E. Kupfer D. J. (2007). Serotonin-1A receptor imaging in recurrent depression: replication and literature review. Nucl. Med. Biol. 34 (7), 865–877. 10.1016/j.nucmedbio.2007.06.008 17921037 PMC2702715

[B24] Du Y. Gao X. R. Peng L. Ge J. F. (2020). Crosstalk between the microbiota-gut-brain axis and depression. Heliyon 6 (6), e04097. 10.1016/j.heliyon.2020.e04097 32529075 PMC7276434

[B25] Ettman C. K. Cohen G. H. Abdalla S. M. Sampson L. Trinquart L. Castrucci B. C. (2022). Persistent depressive symptoms during COVID-19: a national, population-representative, longitudinal study of U.S. adults. Lancet reg. health Am. 5, 100091. 10.1016/j.lana.2021.100091 34635882 PMC8488314

[B26] Eutamene H. Lamine F. Chabo C. Theodorou V. Rochat F. Bergonzelli G. E. (2007). Synergy between Lactobacillus paracasei and its bacterial products to counteract stress-induced gut permeability and sensitivity increase in rats. J. Nutr. 137 (8), 1901–1907. 10.1093/jn/137.8.1901 17634262

[B27] Frémont M. Coomans D. Massart S. De Meirleir K. (2013). High-throughput 16S rRNA gene sequencing reveals alterations of intestinal microbiota in myalgic encephalomyelitis/chronic fatigue syndrome patients. Anaerobe 22, 50–56. 10.1016/j.anaerobe.2013.06.002 23791918

[B28] Frost G. Sleeth M. L. Sahuri-Arisoylu M. Lizarbe B. Cerdan S. Brody L. (2014). The short-chain fatty acid acetate reduces appetite via a central homeostatic mechanism. Nat. Commun. 5, 3611. 10.1038/ncomms4611 24781306 PMC4015327

[B29] Fu Q. Wang P. Zhang Y. Wu T. Huang J. Song Z. (2023). Effects of dietary inclusion of asiaticoside on growth performance, lipid metabolism, and gut microbiota in yellow-feathered chickens. Animals open access J. MDPI 13 (16), 2653. 10.3390/ani13162653 PMC10451259 37627444

[B30] Gilbert J. A. Blaser M. J. Caporaso J. G. Jansson J. K. Lynch S. V. Knight R. (2018). Current understanding of the human microbiome. Nat. Med. 24 (4), 392–400. 10.1038/nm.4517 29634682 PMC7043356

[B31] Goodwin R. D. Dierker L. C. Wu M. Galea S. Hoven C. W. Weinberger A. H. (2022). Trends in U.S. Depression prevalence from 2015 to 2020: the widening treatment gap. Am. J. Prev. Med. 63 (5), 726–733. 10.1016/j.amepre.2022.05.014 36272761 PMC9483000

[B32] Guo L. Ren L. Zhang C. (2018). Relationship between depression and inflammatory factors and brain-derived neurotrophic factor in patients with perimenopause syndrome. Exp. Ther. Med. 15 (5), 4436–4440. 10.3892/etm.2018.5985 29849779 PMC5962862

[B33] Hamon M. Blier P. (2013). Monoamine neurocircuitry in depression and strategies for new treatments. Prog. neuro-psychopharmacology and Biol. psychiatry 45, 54–63. 10.1016/j.pnpbp.2013.04.009 23602950

[B34] Han S. K. Kim J. K. Park H. S. Shin Y. J. Kim D. H. (2021). Chaihu-Shugan-San (Shihosogansan) alleviates restraint stress-generated anxiety and depression in mice by regulating NF-κB-mediated BDNF expression through the modulation of gut microbiota. Chin. Med. 16 (1), 77. 10.1186/s13020-021-00492-5 34391441 PMC8364688

[B35] Hassamal S. (2023). Chronic stress, neuroinflammation, and depression: an overview of pathophysiological mechanisms and emerging anti-inflammatories. Front. psychiatry 14, 1130989. 10.3389/fpsyt.2023.1130989 37252156 PMC10213648

[B36] He Z. Hu Y. Niu Z. Zhong K. Liu T. Yang M. (2023). A review of pharmacokinetic and pharmacological properties of asiaticoside, a major active constituent of *Centella asiatica* (L.) Urb. J. Ethnopharmacol. 302 (Pt A), 115865. 10.1016/j.jep.2022.115865 36306932

[B37] Huang Y. Wang Y. Wang H. Liu Z. Yu X. Yan J. (2019). Prevalence of mental disorders in China: a cross-sectional epidemiological study. lancet Psychiatry 6 (3), 211–224. 10.1016/S2215-0366(18)30511-X 30792114

[B38] Jesulola E. Micalos P. Baguley I. J. (2018). Understanding the pathophysiology of depression: from monoamines to the neurogenesis hypothesis model - are we there yet? Behav. Brain Res. 341, 79–90. 10.1016/j.bbr.2017.12.025 29284108

[B39] Jiang H. Ling Z. Zhang Y. Mao H. Ma Z. Yin Y. (2015). Altered fecal microbiota composition in patients with major depressive disorder. Brain, Behav. Immun. 48, 186–194. 10.1016/j.bbi.2015.03.016 25882912

[B40] Jiang N. Lv J. Wang H. Huang H. Wang Q. Lu C. (2020). Ginsenoside Rg1 ameliorates chronic social defeat stress-induced depressive-like behaviors and hippocampal neuroinflammation. Life Sci. 252, 117669. 10.1016/j.lfs.2020.117669 32298740

[B41] Kang H. J. Bae K. Y. Kim S. W. Shin I. S. Hong Y. J. Ahn Y. (2016). BDNF val66met polymorphism and depressive disorders in patients with acute coronary syndrome. J. Affect. Disord. 194, 1–8. 10.1016/j.jad.2016.01.033 26795846

[B42] Keller J. Gomez R. Williams G. Lembke A. Lazzeroni L. Murphy G. M. Jr. (2017). HPA axis in major depression: cortisol, clinical symptomatology and genetic variation predict cognition. Mol. psychiatry 22 (4), 527–536. 10.1038/mp.2016.120 27528460 PMC5313380

[B43] Kishi T. Yoshimura R. Ikuta T. Iwata N. (2017). Brain-derived neurotrophic factor and major depressive disorder: evidence from meta-analyses. Front. psychiatry 8, 308. 10.3389/fpsyt.2017.00308 29387021 PMC5776079

[B44] Koh A. De Vadder F. Kovatcheva-Datchary P. Bäckhed F. (2016). From dietary fiber to host physiology: short-chain fatty acids as key bacterial metabolites. Cell 165 (6), 1332–1345. 10.1016/j.cell.2016.05.041 27259147

[B45] Li J. Zhou Y. Liu B. B. Liu Q. Geng D. Weng L. J. (2013). Nobiletin ameliorates the deficits in hippocampal BDNF, TrkB, and synapsin I induced by chronic unpredictable mild stress. Evidence-based complementary Altern. Med. eCAM. 2013, 359682. 10.1155/2013/359682 PMC3613093 23573124

[B46] Li Y. Peng Y. Ma P. Yang H. Xiong H. Wang M. (2018). Antidepressant-like effects of cistanche tubulosa extract on chronic unpredictable stress rats through restoration of gut microbiota homeostasis. Front. Pharmacol. 9, 967. 10.3389/fphar.2018.00967 30186183 PMC6112285

[B47] Liang X. Yan Ni H. Si Wei C. Wen J. W. Xu N. Cui S. (2008). Antidepressant-like effect of asiaticoside in mice. Pharmacol. Biochem. Behav. 89 (3), 444–449. 10.1016/j.pbb.2008.01.020 18325568

[B48] Liu P. Wang Y. Yang G. Zhang Q. Meng L. Xin Y. (2021). The role of short-chain fatty acids in intestinal barrier function, inflammation, oxidative stress, and colonic carcinogenesis. Pharmacol. Res. 165, 105420. 10.1016/j.phrs.2021.105420 33434620

[B49] Llopis M. Antolin M. Carol M. Borruel N. Casellas F. Martinez C. (2009). Lactobacillus casei downregulates commensals' inflammatory signals in Crohn's disease mucosa. Inflamm. bowel Dis. 15 (2), 275–283. 10.1002/ibd.20736 18839424

[B50] Luo L. Liu X. L. Mu R. H. Wu Y. J. Liu B. B. Geng D. (2015). Hippocampal BDNF signaling restored with chronic asiaticoside treatment in depression-like mice. Brain Res. Bull. 114, 62–69. 10.1016/j.brainresbull.2015.03.006 25857945

[B51] Ma J. Wang R. Chen Y. Wang Z. Dong Y. (2023). 5-HT attenuates chronic stress-induced cognitive impairment in mice through intestinal flora disruption. J. neuroinflammation 20 (1), 23. 10.1186/s12974-023-02693-1 36737776 PMC9896737

[B52] Madison A. A. Bailey M. T. (2024). Stressed to the core: inflammation and intestinal permeability link stress-related gut microbiota shifts to mental health outcomes. Biol. psychiatry 95 (4), 339–347. 10.1016/j.biopsych.2023.10.014 38353184 PMC10867428

[B53] Markov D. D. (2022). Sucrose preference test as a measure of anhedonic behavior in a chronic unpredictable mild stress model of depression: outstanding issues. Brain Sci. 12 (10), 1287. 10.3390/brainsci12101287 36291221 PMC9599556

[B54] Martel J. Chang S. H. Ko Y. F. Hwang T. L. Young J. D. Ojcius D. M. (2022). Gut barrier disruption and chronic disease. Trends Endocrinol. metabolism TEM 33 (4), 247–265. 10.1016/j.tem.2022.01.002 35151560

[B55] Martin C. R. Osadchiy V. Kalani A. Mayer E. A. (2018). The brain-gut-microbiome Axis. Cell. Mol. gastroenterology hepatology 6 (2), 133–148. 10.1016/j.jcmgh.2018.04.003 PMC6047317 30023410

[B56] McGaughey K. D. Yilmaz-Swenson T. Elsayed N. M. Cruz D. A. Rodriguiz R. M. Kritzer M. D. (2019). Relative abundance of Akkermansia spp. and other bacterial phylotypes correlates with anxiety- and depressive-like behavior following social defeat in mice. Sci. Rep. 9 (1), 3281. 10.1038/s41598-019-40140-5 30824791 PMC6397238

[B57] Menneson S. Ménicot S. Ferret-Bernard S. Guérin S. Romé V. Le Normand L. (2019). Validation of a psychosocial chronic stress model in the pig using a multidisciplinary approach at the gut-brain and behavior levels. Front. Behav. Neurosci. 13, 161. 10.3389/fnbeh.2019.00161 31379533 PMC6646532

[B58] Miller A. H. Raison C. L. (2016). The role of inflammation in depression: from evolutionary imperative to modern treatment target. Nat. Rev. Immunol. 16 (1), 22–34. 10.1038/nri.2015.5 26711676 PMC5542678

[B59] Morais L. H. Schreiber H. L. Mazmanian S. K. (2021). The gut microbiota-brain axis in behaviour and brain disorders. Nat. Rev. Microbiol. 19 (4), 241–255. 10.1038/s41579-020-00460-0 33093662

[B60] Nabavi S. M. Daglia M. Braidy N. Nabavi S. F. (2017). Natural products, micronutrients, and nutraceuticals for the treatment of depression: a short review. Nutr. Neurosci. 20 (3), 180–194. 10.1080/1028415X.2015.1103461 26613119

[B61] Naseribafrouei A. Hestad K. Avershina E. Sekelja M. Linløkken A. Wilson R. (2014). Correlation between the human fecal microbiota and depression. Neurogastroenterol. Motil. 26 (8), 1155–1162. 10.1111/nmo.12378 24888394

[B62] Nestler E. J. Hyman S. E. (2010). Animal models of neuropsychiatric disorders. Nat. Neurosci. 13 (10), 1161–1169. 10.1038/nn.2647 20877280 PMC3750731

[B63] Osadchiy V. Martin C. R. Mayer E. A. (2019). The gut-brain Axis and the microbiome: mechanisms and clinical implications. Clin. gastroenterology hepatology official Clin. Pract. J. Am. Gastroenterological Assoc. 17 (2), 322–332. 10.1016/j.cgh.2018.10.002 PMC6999848 30292888

[B64] Pariante C. M. (2017). Why are depressed patients inflamed? A reflection on 20 years of research on depression, glucocorticoid resistance and inflammation. Eur. Neuropsychopharmacol. J. Eur. Coll. Neuropsychopharmacol. 27 (6), 554–559. 10.1016/j.euroneuro.2017.04.001 28479211

[B65] Pereira S. C. Coeli-Lacchini F. B. Pereira D. A. Ferezin L. P. Menezes I. C. Baes C. V. W. (2024). Early life stress unravels epistatic genetic associations of cortisol pathway genes with depression. J. psychiatric Res. 175, 323–332. 10.1016/j.jpsychires.2024.05.032 38759498

[B66] Pires L. González-Paramás A. M. Heleno S. A. Calhelha R. C. (2024). The role of gut microbiota in the etiopathogenesis of multiple chronic diseases. Antibiot. Basel, Switz. 13 (5), 392. 10.3390/antibiotics13050392 PMC11117238 38786121

[B67] Qiao H. Li M. X. Xu C. Chen H. B. An S. C. Ma X. M. (2016). Dendritic spines in depression: what we learned from animal models. Neural plast. 2016, 8056370. 10.1155/2016/8056370 26881133 PMC4736982

[B68] Rana T. Behl T. Sehgal A. Srivastava P. Bungau S. (2021). Unfolding the role of BDNF as a biomarker for treatment of depression. J. Mol. Neurosci. 71 (10), 2008–2021. 10.1007/s12031-020-01754-x 33230708

[B69] Rincel M. Aubert P. Chevalier J. Grohard P. A. Basso L. Monchaux de Oliveira C. (2019). Multi-hit early life adversity affects gut microbiota, brain and behavior in a sex-dependent manner. Brain, Behav. Immun. 80, 179–192. 10.1016/j.bbi.2019.03.006 30872090

[B70] Rowan F. E. Docherty N. G. Coffey J. C. O'Connell P. R. (2009). Sulphate-reducing bacteria and hydrogen sulphide in the aetiology of ulcerative colitis. Br. J. Surg. 96 (2), 151–158. 10.1002/bjs.6454 19160346

[B71] Ruilian L. Honglin Q. Jun X. Jianxin L. Qingyun B. Yilin C. (2021). H(2)S-mediated aerobic exercise antagonizes the hippocampal inflammatory response in CUMS-depressed mice. J. Affect. Disord. 283, 410–419. 10.1016/j.jad.2021.02.005 33581467

[B72] Samuel B. S. Shaito A. Motoike T. Rey F. E. Backhed F. Manchester J. K. (2008). Effects of the gut microbiota on host adiposity are modulated by the short-chain fatty-acid binding G protein-coupled receptor, Gpr41. Proc. Natl. Acad. Sci. U. S. A. 105 (43), 16767–16772. 10.1073/pnas.0808567105 18931303 PMC2569967

[B73] Saulnier D. M. Riehle K. Mistretta T. A. Diaz M. A. Mandal D. Raza S. (2011). Gastrointestinal microbiome signatures of pediatric patients with irritable bowel syndrome. Gastroenterology 141 (5), 1782–1791. 10.1053/j.gastro.2011.06.072 21741921 PMC3417828

[B74] Smith M. V. Mazure C. M. (2021). Mental health and wealth: depression, gender, poverty, and parenting. Annu. Rev. Clin. Psychol. 17, 181–205. 10.1146/annurev-clinpsy-071219-022710 33962537

[B75] Sokol H. Pigneur B. Watterlot L. Lakhdari O. Bermúdez-Humarán L. G. Gratadoux J. J. (2008). Faecalibacterium prausnitzii is an anti-inflammatory commensal bacterium identified by gut microbiota analysis of Crohn disease patients. Proc. Natl. Acad. Sci. U. S. A. 105 (43), 16731–16736. 10.1073/pnas.0804812105 18936492 PMC2575488

[B76] Sun B. Wu L. Wu Y. Zhang C. Qin L. Hayashi M. (2020). Therapeutic potential of *Centella asiatica* and its triterpenes: a review. Front. Pharmacol. 11, 568032. 10.3389/fphar.2020.568032 33013406 PMC7498642

[B77] Sun H. Wang N. Cang Z. Zhu C. Zhao L. Nie X. (2016). Modulation of microbiota-gut-brain Axis by berberine resulting in improved metabolic status in high-fat diet-fed rats. Obes. facts 9 (6), 365–378. 10.1159/000449507 27898425 PMC5644798

[B78] Tian T. Mao Q. Xie J. Wang Y. Shao W. H. Zhong Q. (2022). Multi-omics data reveals the disturbance of glycerophospholipid metabolism caused by disordered gut microbiota in depressed mice. J. Adv. Res. 39, 135–145. 10.1016/j.jare.2021.10.002 35777903 PMC9263645

[B79] Tillmann S. Abildgaard A. Winther G. Wegener G. (2019). Altered fecal microbiota composition in the Flinders sensitive line rat model of depression. Psychopharmacology 236 (5), 1445–1457. 10.1007/s00213-018-5094-2 30470860 PMC6599185

[B80] van de Wouw M. Boehme M. Lyte J. M. Wiley N. Strain C. O'Sullivan O. (2018). Short-chain fatty acids: microbial metabolites that alleviate stress-induced brain-gut axis alterations. J. physiology 596 (20), 4923–4944. 10.1113/JP276431 PMC6187046 30066368

[B81] Varela R. B. Valvassori S. S. Lopes-Borges J. Mariot E. Dal-Pont G. C. Amboni R. T. (2015). Sodium butyrate and mood stabilizers block ouabain-induced hyperlocomotion and increase BDNF, NGF and GDNF levels in brain of Wistar rats. J. psychiatric Res. 61, 114–121. 10.1016/j.jpsychires.2014.11.003 25467060

[B82] Verstreken I. Laleman W. Wauters G. Verhaegen J. (2012). Desulfovibrio desulfuricans bacteremia in an immunocompromised host with a liver graft and ulcerative colitis. J. Clin. Microbiol. 50 (1), 199–201. 10.1128/JCM.00987-11 22075582 PMC3256723

[B83] Vuong H. E. Yano J. M. Fung T. C. Hsiao E. Y. (2017). The microbiome and host behavior. Annu. Rev. Neurosci. 40, 21–49. 10.1146/annurev-neuro-072116-031347 28301775 PMC6661159

[B84] Wang L. Guo T. Guo Y. Xu Y. (2020). Asiaticoside produces an antidepressant-like effect in a chronic unpredictable mild stress model of depression in mice, involving reversion of inflammation and the PKA/pCREB/BDNF signaling pathway. Mol. Med. Rep. 22 (3), 2364–2372. 10.3892/mmr.2020.11305 32705202 PMC7411460

[B85] Willner P. (1997). Validity, reliability and utility of the chronic mild stress model of depression: a 10-year review and evaluation. Psychopharmacology 134 (4), 319–329. 10.1007/s002130050456 9452163

[B86] Xiao S. Jiang S. Qian D. Duan J. (2020). Modulation of microbially derived short-chain fatty acids on intestinal homeostasis, metabolism, and neuropsychiatric disorder. Appl. Microbiol. Biotechnol. 104 (2), 589–601. 10.1007/s00253-019-10312-4 31865438

[B87] Yang J. Zheng P. Li Y. Wu J. Tan X. Zhou J. (2020). Landscapes of bacterial and metabolic signatures and their interaction in major depressive disorders. Sci. Adv. 6 (49), eaba8555. 10.1126/sciadv.aba8555 33268363 PMC7710361

[B88] Yang P. Huang S. Luo Z. Zhou S. Zhang C. Zhu Y. (2024). Radix Bupleuri aqueous extract attenuates MK801-induced schizophrenia-like symptoms in mice: participation of intestinal flora. Biomed. and Pharmacother. = Biomedecine and Pharmacother. 172, 116267. 10.1016/j.biopha.2024.116267 38364739

[B89] Yi L. T. Li J. Liu B. B. Luo L. Liu Q. Geng D. (2014). BDNF-ERK-CREB signalling mediates the role of miR-132 in the regulation of the effects of oleanolic acid in male mice. J. psychiatry and Neurosci. JPN 39 (5), 348–359. 10.1503/jpn.130169 25079084 PMC4160364

[B90] Yohn C. N. Gergues M. M. Samuels B. A. (2017). The role of 5-HT receptors in depression. Mol. Brain 10 (1), 28. 10.1186/s13041-017-0306-y 28646910 PMC5483313

[B91] Yun S. W. Park H. S. Shin Y. J. Ma X. Han M. J. Kim D. H. (2023). Lactobacillus gasseri NK109 and its supplement alleviate cognitive impairment in mice by modulating NF-κB activation, BDNF expression, and gut microbiota composition. Nutrients 15 (3), 790. 10.3390/nu15030790 36771498 PMC9921112

[B92] Zhang K. Wang F. Zhai M. He M. Hu Y. Feng L. (2023). Hyperactive neuronal autophagy depletes BDNF and impairs adult hippocampal neurogenesis in a corticosterone-induced mouse model of depression. Theranostics 13 (3), 1059–1075. 10.7150/thno.81067 36793868 PMC9925310

[B93] Zhao Z. X. Fu J. Ma S. R. Peng R. Yu J. B. Cong L. (2018). Gut-brain axis metabolic pathway regulates antidepressant efficacy of albiflorin. Theranostics 8 (21), 5945–5959. 10.7150/thno.28068 30613273 PMC6299426

[B94] Zhu H. Z. Liang Y. D. Ma Q. Y. Hao W. Z. Li X. J. Wu M. S. (2019). Xiaoyaosan improves depressive-like behavior in rats with chronic immobilization stress through modulation of the gut microbiota. Biomed. and Pharmacother. = Biomedecine and Pharmacother. 112, 108621. 10.1016/j.biopha.2019.108621 30798141

