# Supplementary material for: Asiaticoside improves depressive-like behavior in mice with chronic unpredictable mild stress through modulation of the gut microbiota
Source: Front Pharmacol. 2024 Oct 18;15:1461873. doi: 10.3389/fphar.2024.1461873 (PMC11527651; doi:10.3389/fphar.2024.1461873)
Supplement: Supplementary file 1 [file DataSheet1.docx]

**Supplementary Material**


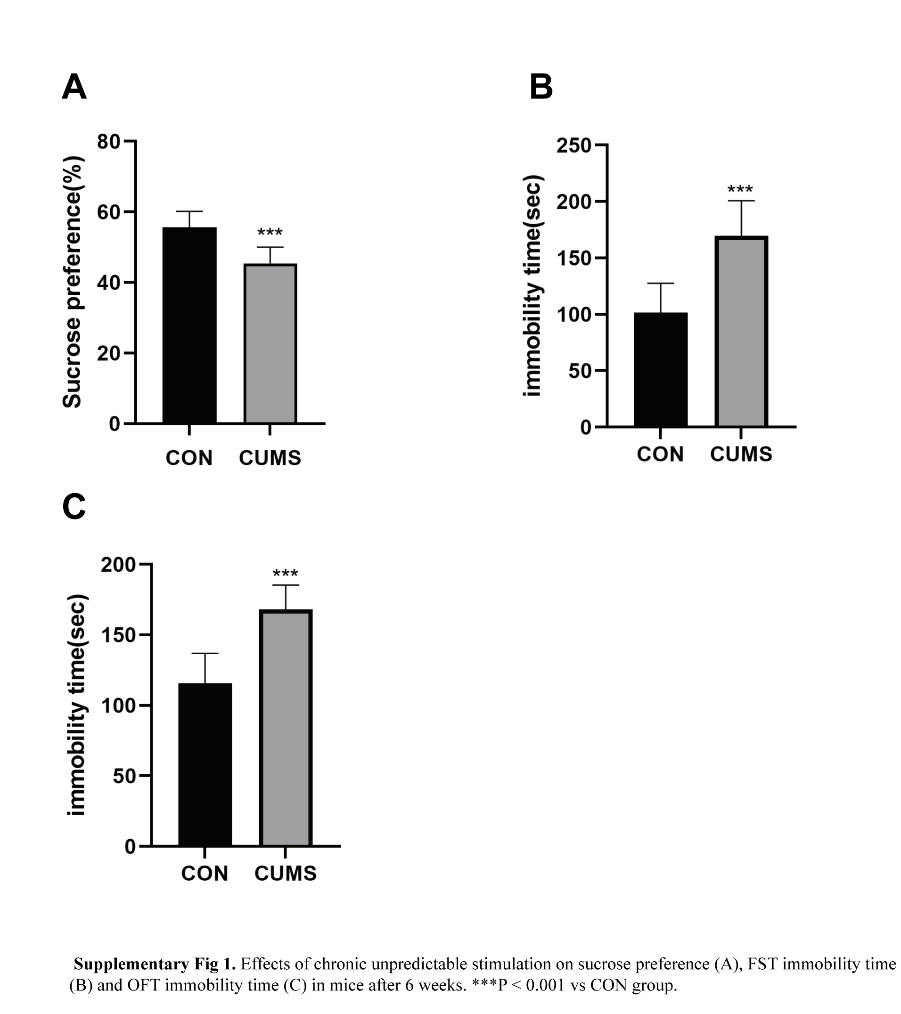


**Supplementary Fig 1.** Effects of chronic unpredictable stimulation on sucrose preference (A), FST immobility time (B) and OFT immobility time (C) in mice after 6 weeks. ***P < 0.001 vs CON group.

**Supplementary Table 1 (See Fig 1.) Information of valid reads of 30 samples in this study.**

| **Sample** | **Sequence number** | **Base number** | **Mean length** | **Minimal length** | **Maximal length** |
| --- | --- | --- | --- | --- | --- |
| CUMS1 | 89567 | 37501024 | 418.6924 | 231 | 535 |
| CUMS2 | 88211 | 37010626 | 419.5693 | 259 | 509 |
| CUMS3 | 90078 | 37557005 | 416.9387 | 273 | 470 |
| CUMS4 | 90182 | 37547393 | 416.3513 | 283 | 502 |
| CUMS5 | 100452 | 42170045 | 419.8029 | 248 | 462 |
| Con1 | 87922 | 36779664 | 418.3215 | 249 | 509 |
| Con2 | 90686 | 38097271 | 420.1009 | 278 | 511 |
| Con3 | 86955 | 36611266 | 421.0369 | 252 | 444 |
| Con4 | 82981 | 34519952 | 415.9983 | 216 | 441 |
| Con5 | 86254 | 35928148 | 416.5389 | 204 | 485 |
| ASL1 | 96052 | 40032342 | 416.7778 | 269 | 436 |
| ASL2 | 89510 | 37642948 | 420.5446 | 277 | 462 |
| ASL3 | 88034 | 36783160 | 417.829 | 267 | 492 |
| ASL4 | 84565 | 35541621 | 420.2876 | 265 | 470 |
| ASL5 | 108942 | 45558618 | 418.1915 | 216 | 443 |
| ASM1 | 88712 | 37434067 | 421.973 | 292 | 442 |
| ASM2 | 83845 | 35598885 | 424.5797 | 252 | 523 |
| ASM3 | 92054 | 38598640 | 419.3043 | 231 | 515 |
| ASM4 | 92956 | 38873825 | 418.196 | 230 | 509 |
| ASM5 | 89419 | 37797896 | 422.7054 | 213 | 437 |
| ASH1 | 85476 | 35885803 | 419.8348 | 269 | 490 |
| ASH2 | 79819 | 33940632 | 425.22 | 262 | 437 |
| ASH3 | 79154 | 33709770 | 425.8758 | 277 | 526 |
| ASH4 | 76168 | 32379794 | 425.1102 | 203 | 434 |
| ASH5 | 90428 | 37890547 | 419.0134 | 235 | 514 |
| FLX1 | 89309 | 37403055 | 418.805 | 278 | 438 |
| FLX2 | 94227 | 39559925 | 419.8364 | 245 | 532 |
| FLX3 | 94008 | 39212938 | 417.1234 | 252 | 510 |
| FLX4 | 93623 | 39291928 | 419.6824 | 249 | 437 |
| FLX5 | 94270 | 39277955 | 416.6538 | 279 | 478 |


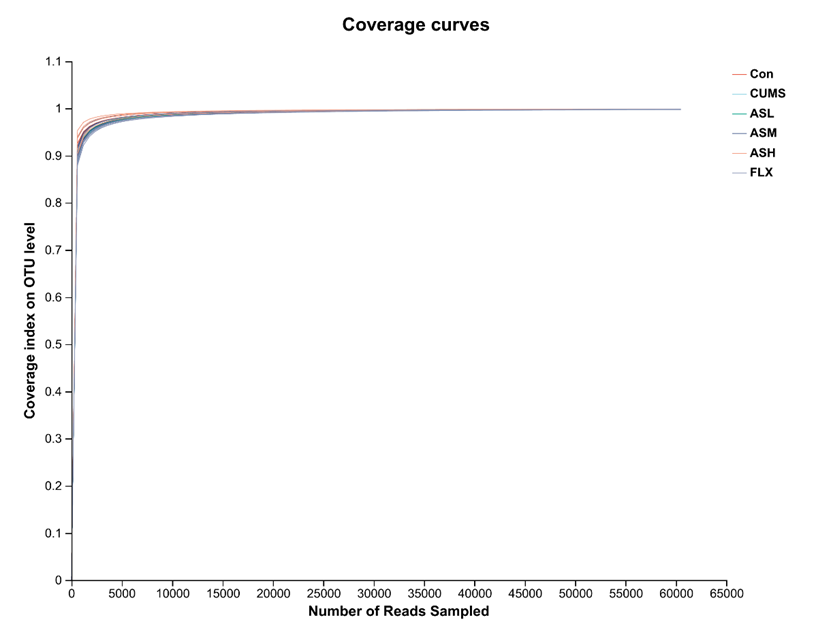


**Supplementary Fig 2.** Rarefaction curves of the different samples performed high-throughput sequencing. The curve tended to be flat and saturated, indicating that the sequence detection range had covered most microorganisms in the sample.
